# Optimization of the NRCS Sampling at the Sea Wind Retrieval by the Airborne Rotating-Beam Scatterometer Mounted under Fuselage

**DOI:** 10.3390/s22114016

**Published:** 2022-05-25

**Authors:** Alexey Nekrasov, Alena Khachaturian, Evgenii Vorobev

**Affiliations:** 1 Department of Radio Engineering Systems, Saint Petersburg Electrotechnical University, Professora Popova 5F, 197022 Saint Petersburg, Russia; khachaturyan.al@gmail.com (A.K.); vorobuevgeniy@gmail.com (E.V.); 2 Institute for Computer Technologies and Information Security, Southern Federal University, Chekhova 2, 347922 Taganrog, Russia

**Keywords:** radar, airborne scatterometer, radar backscatter, sea surface, sea wind retrieval

## Abstract

The optimization of normalized radar cross-section (NRCS) sampling by a scatterometer allows an increase in the accuracy of the wind retrieval over the water surface and a decrease in the time of the measurement. Here, we investigate the possibility of improving wind vector measurement with an airborne rotating-beam scatterometer mounted under the fuselage. For this purpose, we investigated NRCS sampling at various incidence angles, and the possibility of using NRCS samples obtained during simultaneous measurement at different incidence angles to perform wind retrieval. The proposed wind algorithms are based on a geophysical model function (GMF). Sea wind retrieval was carried out using Monte Carlo simulations with consideration of a single incidence angle or combinations of several incidence angles. The incidence angles of interest were 30°, 35°, 40°, 45°, 50°, 55°, and 60°. The simulation showed that the wind speed error decreased with an increase in the incidence angle, and the wind direction error tended to decrease with an increase in the incidence angle. The single incidence angle case is characterized by higher maximum wind retrieval errors but allows for a higher maximum altitude of the wind retrieval method’s applicability to be achieved. The use of several neighboring incidence angles allows a better wind vector retrieval accuracy to be achieved. The combinations of three and four incidence angles provided the lowest maximum wind speed and direction errors in the range of the incidence angles from 45° to 60° but, unfortunately, provide the lowest maximum altitude of applicability of the wind retrieval method. At the same time, the combination of two incidence angles is characterized by slightly higher maximum wind retrieval errors than in the cases of three and four incidence angles, but they are lower than in the case of the single incidence angle. Moreover, the two incidence angles’ combination is a simpler way to decrease the wind retrieval errors, especially for measurement near an incidence angle of 30°, providing nearly the highest maximum altitude of the wind retrieval method applicability. The results obtained can be used to enhance existing airborne radars and in the development of new remote sensing systems.

## 1. Introduction

During the last decades, sea-surface backscattering has been of great interest to researchers. This interest is motivated by the need for a better understanding of sea-surface backscattering as a physical phenomenon and by its prospective application in the development and improvement of remote sensing technology. Therefore, research on sea-surface backscattering is very important to understanding the formation mechanism of sea clutter, which is crucial for radar target detection in nonhomogeneous environments [1,2,3,4], and for operational monitoring of waves, currents, and sea winds [5,6,7].

Water backscattering is studied by means of a sensor called a scatterometer. Experiments have been performed in wind-wave tanks [7,8], on sea platforms [9,10], and by airborne [11,12] and spaceborne [13,14] scatterometers. The identified relationship between the backscatter and wind vector over sea made it possible to use scatterometers for remote measurement of the wind vector over water surfaces [15].

Near-surface wind retrieval is performed with a wind algorithm. The wind algorithm is based on a GMF and takes into account the specificity of the measuring geometry of a scatterometer [16].

Scatterometers placed on one or several satellites provide current information about the wind conditions over oceans and seas at a global scale. At the same time, scatterometers’ placement on aircraft allows local information on the wind over water to be obtained, which can clarify the information received from satellites for meteorological and navigation applications and for scientific purposes.

Airborne scatterometers (or multimode radars with a scatterometer mode) have a fixed-beam antenna [17,18,19,20], scanning antenna [21,22,23], or rotating-beam antenna [12,24,25,26,27,28]. Mostly, antennas rotating in the horizontal plane are installed on the bottom or under the fuselage.

Scatterometers with a fixed-beam antenna require the measurements to be on a circular ground track [19,20,29]. On the contrary, scatterometers with scanning [30,31,32] or rotating-beam [12,27] antennas require the measurements to be on a rectilinear ground track.

Airborne sea-wind measurements using rotating-beam scatterometers has quite a long heritage. The prime examples of such scatterometers are KU-SCAT (Ku-band scatterometer) and C-SCAT (C-band scatterometer) of the Microwave Remote Sensing Laboratory at the University of Massachusetts Amherst [12], DUTSCAT (multifrequency Delft University of Technology Scatterometer) [33], RACS (German Rotating Antenna C-band Scatterometer) [11], IWRAP (C- and Ka-band Imaging Wind and Rain Airborne Profiler) of the Microwave Remote Sensing Laboratory at the University of Massachusetts Amherst [24], and DopplerScatt (Ka-band pencil-beam Doppler scatterometer) of the NASA Instrument Incubator Program [6].

**Figure 1:**
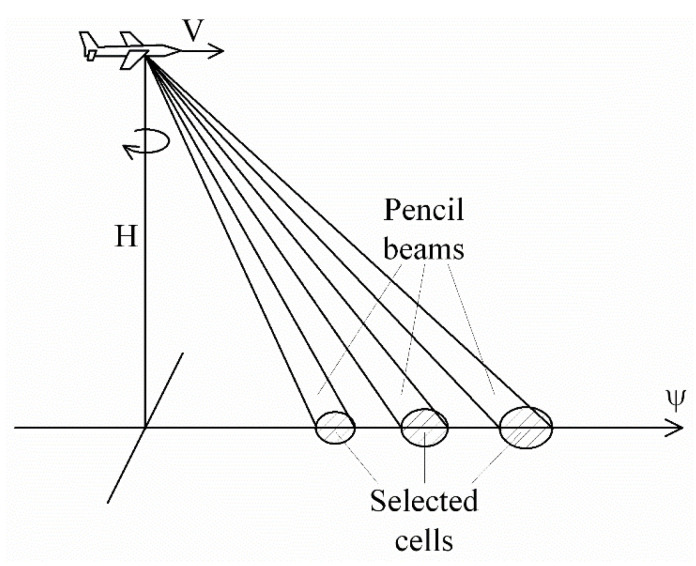
Rotating antenna multibeam geometry (three-beam case in the vertical plane): *V* is the speed of flight; *H* is the altitude; *ψ* is the aircraft flight direction.

**Figure 2:**
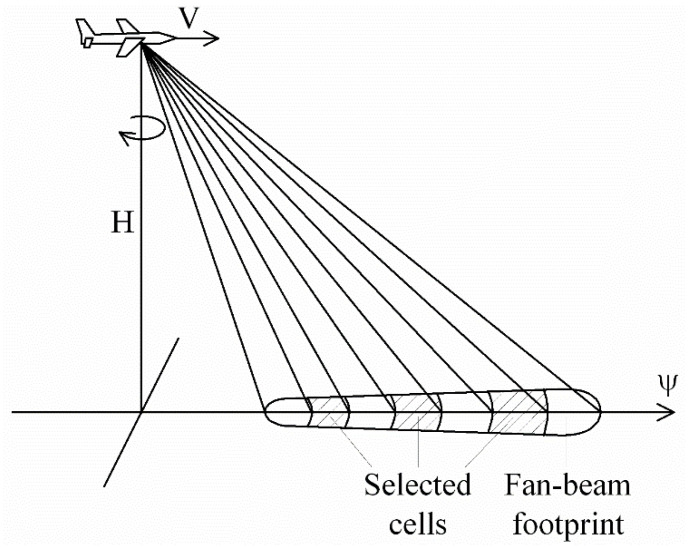
Rotating antenna fan-beam geometry (three selected sell case in the vertical plane): *V* is the speed of flight; *H* is the altitude; *ψ* is the aircraft flight direction.

In the case of airborne scatterometers, the rotating antenna has one or several pencil beams (Figure 1), or a fan beam (Figure 2) [24,34,35]. Multiple beams located in the same vertical plane at different incidence angles allow the measured NRCSs (simultaneously) to be obtained at all their incidence angles. A similar capability is shown by the fan beam when time-delay selection is applied.

Usually, only one incidence angle is used for wind retrieval by an airborne scatterometer with a rotating antenna. However, a multibeam or fan-beam geometry can achieve NRCS sampling at several incidence angles in the same vertical plane. In this connection, this study was motivated by the need for an enhancement in the functionality of radars with such observation geometries and further increases in the wind retrieval accuracy. The simultaneous use of the measured NRCSs at several incidence angles in the same plane seems promising for wind measurement by airborne scatterometers (or multimode radars with the scatterometer mode) mounted under the fuselage. Thus, this manuscript addresses the analysis of such geometries and their possible implementation for better wind retrieval over the sea, e.g., with airborne scatterometers or enhanced airborne maritime/ground surveillance radars.

Section 2 introduces the background of wind retrieval using a scatterometer and the wind retrieval algorithms developed to estimate the wind vector over the sea by airborne scatterometers with a rotating antenna sampling NRCSs at a single incidence angle or combinations of several incidence angles. Section 3 describes the simulations, presents the results obtained and their discussion, and suggestions for future research. Finally, the conclusions are outlined in Section 4.

## 2. Materials and Methods

A wind scatterometer is an airborne or spaceborne microwave sensor designed for operational measurement of the wind vector over the ocean or sea [7]. The wind vector retrieval by a scatterometer depends on NRCSs sampling from different azimuthal directions (and different or the same incidence angles depending on the scatterometer configuration and its installation on an aircraft or satellite) and a water GMF representing the NRCS $\sigma^{\circ }(U,\theta ,\alpha )$ dependence on the wind speed *U*, incidence angle *θ*, and azimuthal angle *α* relative to the up-wind direction. The GMFs are described in various analytical forms and can be presented only as a table. One such analytical GMF form is as follows [36]:

(1) $$\sigma^{\circ }(U,\theta ,\alpha )=A(U,\theta )+B(U,\theta )\cos\alpha +C(U,\theta )\cos(2\alpha ),$$

where A(U,θ), B(U,θ), and C(U,θ) are the coefficients written as $A(U,\theta )=a_{0}(\theta )U^{\gamma_{0}(\theta )}$, $B(U,\theta )=a_{1}(\theta )U^{\gamma_{1}(\theta )}$, and $C(U,\theta )=a_{2}(\theta )U^{\gamma_{2}(\theta )}$; $a_{0}(\theta )$, $a_{1}(\theta )$, $a_{2}(\theta )$, $\gamma_{0}(\theta )$, $\gamma_{1}(\theta )$, and $\gamma_{2}(\theta )$ are the coefficients corresponding to the appropriate incidence angle, radar wavelength, and polarization.

In the general case, wind vector retrieval by an airborne scatterometer with a rotating antenna that samples NRCSs at one incidence angle only can be achieved by solving the system of *N* equations [28,37]:

(2) $$\left\{\begin{array}{l} \sigma^{\circ }(U,\theta ,\alpha +\psi_{1})=A(U,\theta )\;+B(U,\theta )\cos(\alpha +\psi_{1})+C(U,\theta )\cos(2(\alpha +\psi_{1})),\\ \cdots \cdots \cdots \cdots \cdots \cdots \cdots \cdots \cdots \cdots \cdots \cdots \cdots \cdots \cdots \cdots \cdots \cdots \cdots \cdots \cdots \cdots \cdots \cdots \cdots \cdots \\ \sigma^{\circ }(U,\theta ,\alpha +\psi_{i})=A(U,\theta )+B(U,\theta )\cos(\alpha +\psi_{i})+C(U,\theta )\cos(2(\alpha +\psi_{i})),\\ \cdots \cdots \cdots \cdots \cdots \cdots \cdots \cdots \cdots \cdots \cdots \cdots \cdots \cdots \cdots \cdots \cdots \cdots \cdots \cdots \cdots \cdots \cdots \cdots \cdots \cdots \\ \sigma^{\circ }(U,\theta ,\alpha +\psi_{N})=A(U,\theta )+B(U,\theta )\cos(\alpha +\psi_{N})+C(U,\theta )\cos(2(\alpha +\psi_{N})), \end{array}\right.$$

where $i=\vec{1,N}$, *N* is the number of the azimuth sectors observed during a whole 360° azimuth observation, $N=360^{\circ }/\Delta \alpha_{s}$; Δ*α_s_* is the angular width of each azimuth sector (composing whole 360° azimuth NRCS curve); $\sigma^{\circ }(U,\theta ,\alpha +\psi_{i})$ is the measured NRCS corresponding to the azimuth sector number *I*; and *ψ_i_* is the direction of the azimuth sector number *i* relative to the aircraft flight direction *ψ*. The system of Equation (2) or similar systems of equations for wind retrieval over water are composed based on GMF Equation (1) under the condition of a narrow antenna beam in the azimuth plane, where the azimuth sector angular width is 15–20° [38,39].

As the system of Equation (2) provides the up-wind direction retrieval, it is converted to the measured wind direction *ψ_w_* using the following equation [40]:

(3) $$\psi_{w}=\psi -\alpha \pm 180^{\circ }.$$

In the case of an airborne scatterometer with a multibeam or fan-beam rotating antenna, it can provide simultaneous NRCS sampling at several incidence angles in the same vertical plane, which seems more advantageous compared to NRCS sampling at only one incidence angle. Thus, the following system of equations can be used for wind retrieval:

(4) $$\left\{\begin{array}{cc} \; & \sigma^{\circ }\left(U,\theta_{1},\alpha +\psi_{1}\right)=A\left(U,\theta_{1}\right)+B\left(U,\theta_{1}\right)\cos\left(\alpha +\psi_{1}\right)+C\left(U,\theta_{1}\right)\cos\left(2\left(\alpha +\psi_{1}\right)\right),\\ \; & \cdots \cdots \cdots \cdots \cdots \cdots \cdots \cdots \cdots \cdots \cdots \cdots \cdots \cdots \cdots \cdots \cdots \cdots \cdots \cdots \cdots \cdots \cdots \cdots \cdots \cdots \cdots \cdots \cdots \cdots \\ \; & \sigma^{\circ }\left(U,\theta_{1},\alpha +\psi_{i}\right)=A\left(U,\theta_{1}\right)+B\left(U,\theta_{1}\right)\cos\left(\alpha +\psi_{i}\right)+C\left(U,\theta_{1}\right)\cos\left(2\left(\alpha +\psi_{i}\right)\right),\\ \; & \cdots \cdots \cdots \cdots \cdots \cdots \cdots \cdots \cdots \cdots \cdots \cdots \cdots \cdots \cdots \cdots \cdots \cdots \cdots \cdots \cdots \cdots \cdots \cdots \cdots \cdots \cdots \cdots \cdots \cdots \\ \; & \sigma^{\circ }\left(U,\theta_{1},\alpha +\psi_{N}\right)=A\left(U,\theta_{1}\right)+B\left(U,\theta_{1}\right)\cos\left(\alpha +\psi_{N}\right)+C\left(U,\theta_{1}\right)\cos\left(2\left(\alpha +\psi_{N}\right)\right),\\ \; & \cdots \cdots \cdots \cdots \cdots \cdots \cdots \cdots \cdots \cdots \cdots \cdots \cdots \cdots \cdots \cdots \cdots \cdots \cdots \cdots \cdots \cdots \cdots \cdots \cdots \cdots \cdots \cdots \cdots \cdots \\ \; & \sigma^{\circ }\left(U,\theta_{j},\alpha +\psi_{1}\right)=A\left(U,\theta_{j}\right)+B\left(U,\theta_{j}\right)\cos\left(\alpha +\psi_{1}\right)+C\left(U,\theta_{j}\right)\cos\left(2\left(\alpha +\psi_{1}\right)\right),\\ \; & \cdots \cdots \cdots \cdots \cdots \cdots \cdots \cdots \cdots \cdots \cdots \cdots \cdots \cdots \cdots \cdots \cdots \cdots \cdots \cdots \cdots \cdots \cdots \cdots \cdots \cdots \cdots \cdots \cdots \cdots \\ \; & \sigma^{\circ }\left(U,\theta_{j},\alpha +\psi_{i}\right)=A\left(U,\theta_{j}\right)+B\left(U,\theta_{j}\right)\cos\left(\alpha +\psi_{i}\right)+C\left(U,\theta_{j}\right)\cos\left(2\left(\alpha +\psi_{i}\right)\right),\\ \; & \cdots \cdots \cdots \cdots \cdots \cdots \cdots \cdots \cdots \cdots \cdots \cdots \cdots \cdots \cdots \cdots \cdots \cdots \cdots \cdots \cdots \cdots \cdots \cdots \cdots \cdots \cdots \cdots \cdots \cdots \\ \; & \sigma^{\circ }\left(U,\theta_{j},\alpha +\psi_{N}\right)=A\left(U,\theta_{j}\right)+B\left(U,\theta_{j}\right)\cos\left(\alpha +\psi_{N}\right)+C\left(U,\theta_{j}\right)\cos\left(2\left(\alpha +\psi_{N}\right)\right),\\ \; & \cdots \cdots \cdots \cdots \cdots \cdots \cdots \cdots \cdots \cdots \cdots \cdots \cdots \cdots \cdots \cdots \cdots \cdots \cdots \cdots \cdots \cdots \cdots \cdots \cdots \cdots \cdots \cdots \cdots \cdots \\ \; & \sigma^{\circ }\left(U,\theta_{K},\alpha +\psi_{1}\right)=A\left(U,\theta_{K}\right)+B\left(U,\theta_{K}\right)\cos\left(\alpha +\psi_{1}\right)+C\left(U,\theta_{K}\right)\cos\left(2\left(\alpha +\psi_{1}\right)\right),\\ \; & \cdots \cdots \cdots \cdots \cdots \cdots \cdots \cdots \cdots \cdots \cdots \cdots \cdots \cdots \cdots \cdots \cdots \cdots \cdots \cdots \cdots \cdots \cdots \cdots \cdots \cdots \cdots \cdots \cdots \cdots \\ \; & \sigma^{\circ }\left(U,\theta_{K},\alpha +\psi_{i}\right)=A\left(U,\theta_{K}\right)+B\left(U,\theta_{K}\right)\cos\left(\alpha +\psi_{i}\right)+C\left(U,\theta_{K}\right)\cos\left(2\left(\alpha +\psi_{i}\right)\right),\\ \; & \cdots \cdots \cdots \cdots \cdots \cdots \cdots \cdots \cdots \cdots \cdots \cdots \cdots \cdots \cdots \cdots \cdots \cdots \cdots \cdots \cdots \cdots \cdots \cdots \cdots \cdots \cdots \cdots \cdots \cdots \\ \; & \sigma^{\circ }\left(U,\theta_{K},\alpha +\psi_{N}\right)=A\left(U,\theta_{K}\right)+B\left(U,\theta_{K}\right)\cos\left(\alpha +\psi_{N}\right)+C\left(U,\theta_{K}\right)\cos\left(2\left(\alpha +\psi_{N}\right)\right), \end{array}\right.$$

where $j=\vec{1,K}$, *K* is the number of the incidence angles observed (or used for wind retrieval in the case of a multibeam or fan-beam antenna rotating in the horizontal plane), $\sigma^{\circ }(U,\theta_{j},\alpha +\psi_{i})$ is the measured NRCS corresponding to incidence angle number *j*, and azimuth sector number *i*. The system of Equation (4) is also composed based on GMF Equation (1) for each azimuth sector observed during the whole 360° azimuth observation at each incidence angle of interest under the conditions of a narrow antenna beam in the azimuth plane, where the azimuth sector angular width is 15–20° [38,39].

The GMF form of Equation (1) has a particular feature in that the azimuthally averaged NRCS at the same incidence angle $\sigma_{av\;360^{\circ }}^{\circ }(U,\theta )$ can be written as [41]:

(5) $$\sigma_{av\;360^{\circ }}^{\circ }(U,\theta )=\frac{1}{N}\sum_{i=1}^{N}\sigma^{\circ }(U,\theta ,\alpha +\psi_{i})=A(U,\theta )=a_{0}(\theta )U^{\gamma_{0}(\theta )},$$

and this feature can be applied to simplify and speed up the wind speed estimation procedure using a modified system of the equation obtained from the system of Equation (4) with the help of Equation (5):

(6) $$\left\{\begin{array}{l} \sigma_{av\;360^{\circ }}^{\circ }(U,\theta_{1})=a_{0}(\theta_{1})U^{\gamma_{0}(\theta_{1})},\\ \cdots \cdots \cdots \cdots \cdots \cdots \cdots \cdots \cdots \cdots \\ \sigma_{av\;360^{\circ }}^{\circ }(U,\theta_{j})=a_{0}(\theta_{j})U^{\gamma_{0}(\theta_{j})}\\ \cdots \cdots \cdots \cdots \cdots \cdots \cdots \cdots \cdots \cdots \\ \sigma_{av\;360^{\circ }}^{\circ }(U,\theta_{K})=a_{0}(\theta_{K})U^{\gamma_{0}(\theta_{K})}. \end{array}\right.$$

Then, the wind direction is calculated using the system of Equations (3) and (4).

Thus, in the case of an airborne rotating-antenna scatterometer with a multibeam or fan-beam antenna geometry installed at the bottom or under an aircraft, simultaneous NRCS sampling at several incidence angles can be used to recover the wind vector over water surfaces.

## 3. Results and Discussion

To evaluate the proposed wind retrieval algorithm and optimize the wind retrieval procedure, we investigated NRCS sampling at various incidence angles and the possibility of using the NRCS samples obtained during simultaneous measurements at different incidence angles with the help of the wind algorithm proposed in Section 2.

For this purpose, we completed Monte Carlo simulations using a Rayleigh power (exponential) distribution and a GMF from Equation (1), with the Ku-band coefficients corresponding to the horizontal polarization [42]:

(7) $$a_{0}(\theta )=10^{2.47324-0.22478\theta +0.001499\theta^{2}},\;a_{1}(\theta )=10^{-0.50593-0.11694\theta +0.000484\theta^{2}},\;a_{2}(\theta )=10^{1.63685-0.2100488\theta +0.001383\theta^{2}},\;\gamma_{0}(\theta )=-0.15+0.071\theta -0.0004\theta^{2},\;\gamma_{1}(\theta )=-0.02+0.061\theta -0.0003\theta^{2},\;\gamma_{2}(\theta )=-0.16+0.074\theta -0.0004\theta^{2}.$$

The incidence angles of interest were 30°, 35°, 40°, 45°, 50°, 55°, and 60°. The whole 360° azimuth circles observed were divided into *N* = 72 azimuth sectors, which provided an azimuth sector width of 5°. In total, 87 “measured” NRCS samples, under the assumption of a 0.2 dB instrumental noise, were generated for each azimuthal sector and each incidence angle of interest. Wind retrieval was performed at wind speeds of 2 to 30 m/s during various combinations of the incidence angles to evaluate their potential and the accuracy of wind vector retrieval. For each combination of wind speed and azimuth angle at each incidence angle of interest, 30 independent trials were performed.

**Figure 3:**
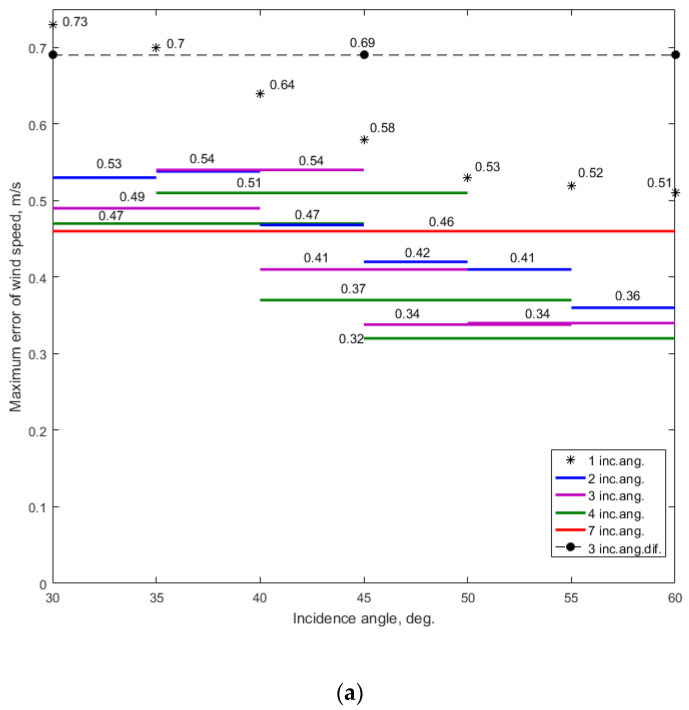

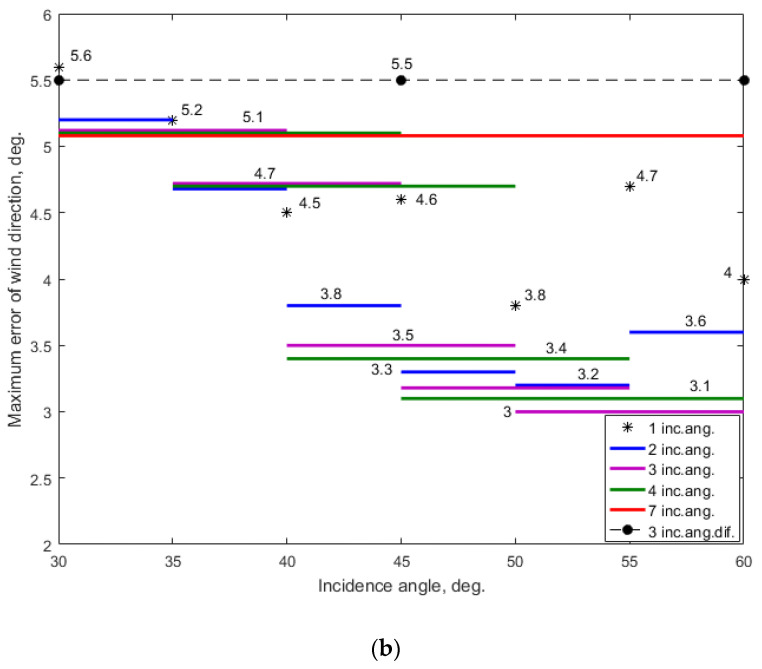
Comparative results for the maximum wind retrieval errors in accordance with the cases considered: (**a**) maximum error of the wind speed; (**b**) maximum error of the wind direction. The black asterisks represent the wind retrieval when one incidence angle was used; the blue lines represent the wind retrieval when two incidence angles were used; the purple lines represent the wind retrieval when three incidence angles were used; the green lines represent the wind retrieval when four incidence angles were used; the red line represents the wind retrieval when seven incidence angles were used; the black dashed line with dots represents the wind retrieval when three incidence angles were used but with a 15° incidence angle difference between the neighboring incidence angles in the range of considered incidence angles of 30° to 60°.

**Figure A1:**
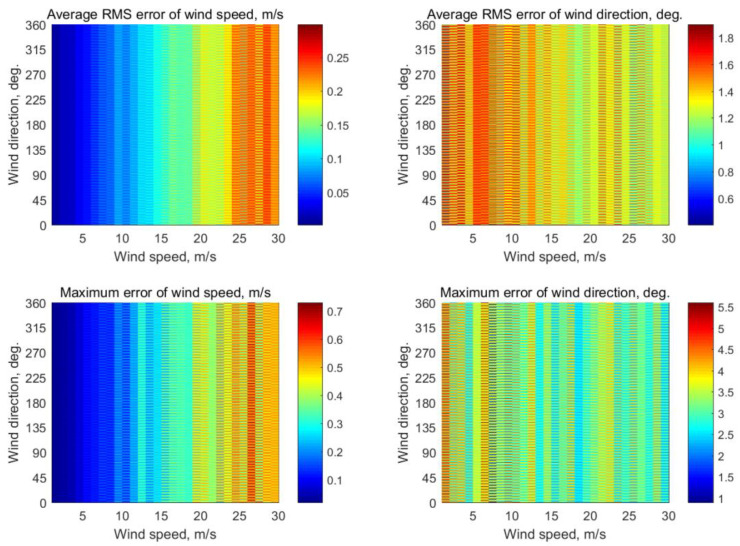
Simulation results of the use of only one incidence angle of 30° for wind retrieval.

**Figure A2:**
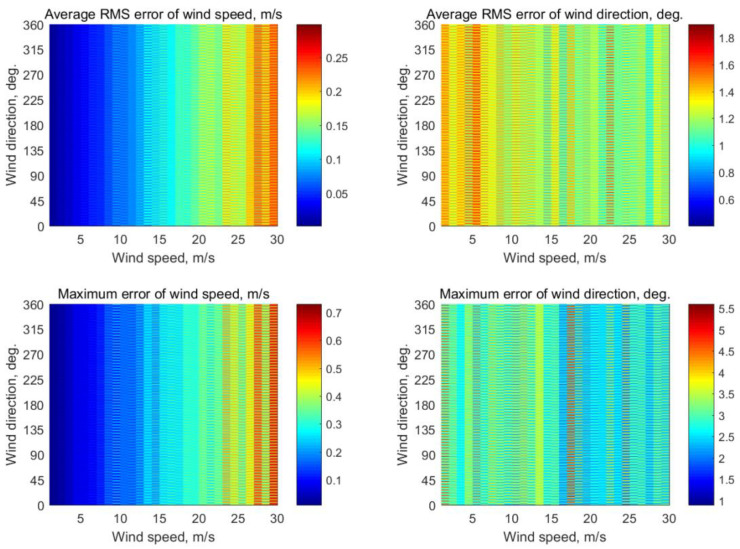
Simulation results of the use of only one incidence angle of 35° for wind retrieval.

**Figure A3:**
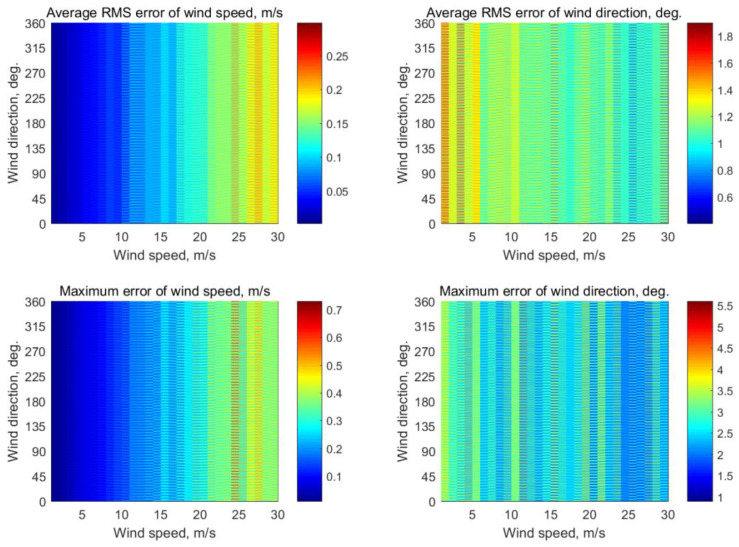
Simulation results of the use of only one incidence angle of 40° for wind retrieval.

**Figure A4:**
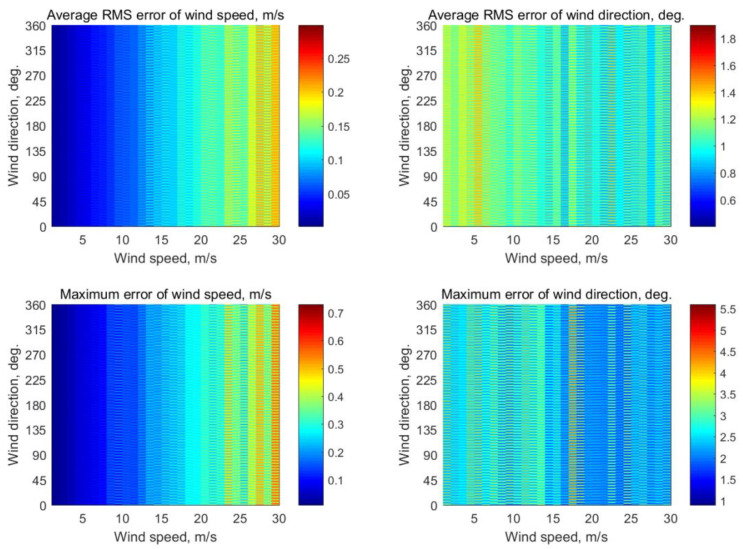
Simulation results of the use of only one incidence angle of 45° for wind retrieval.

**Figure A5:**
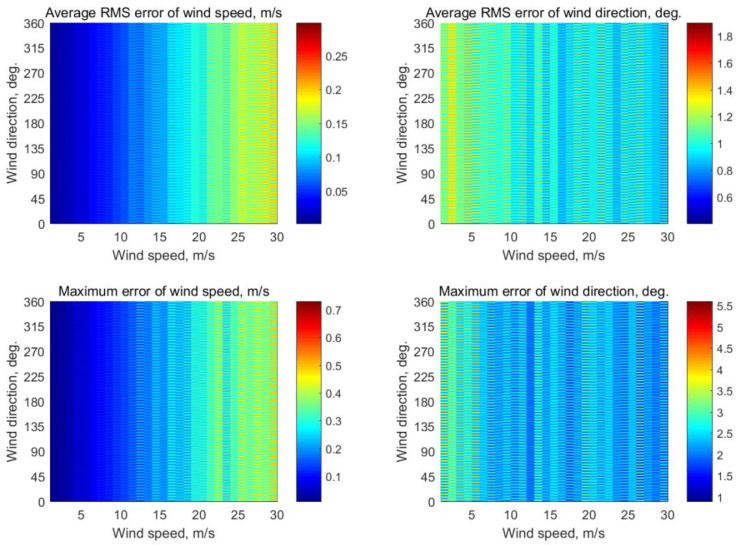
Simulation results of the use of only one incidence angle of 50° for wind retrieval.

**Figure A6:**
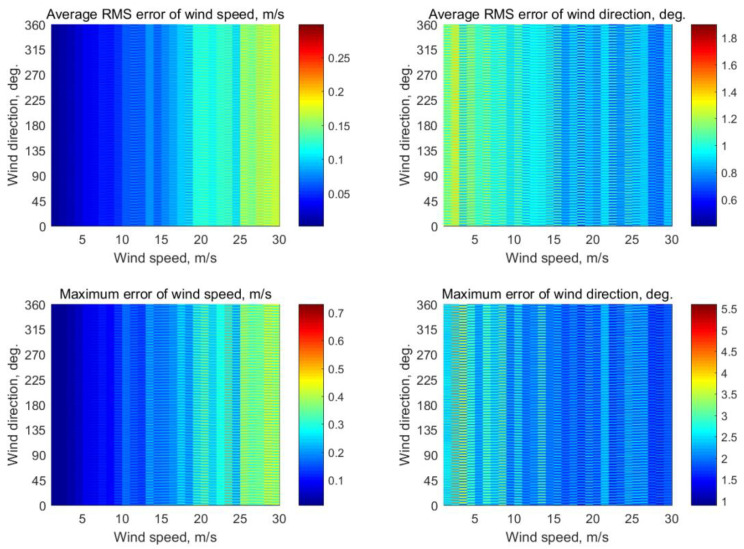
Simulation results of the use of only one incidence angle of 55° for wind retrieval.

**Figure A7:**
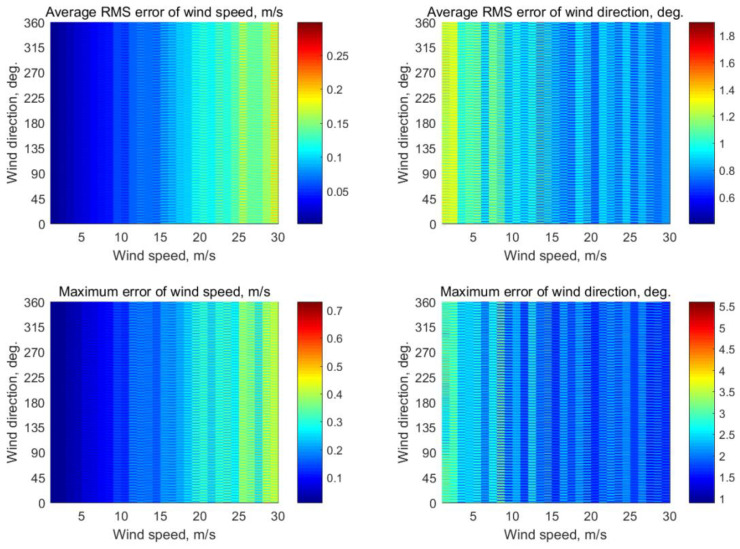
Simulation results of the use of only one incidence angle of 60° for wind retrieval.

First, we evaluated the maximum errors of the wind speed and direction retrieval when only one incidence angle was used. The system of Equation (2) was used for this purpose in the simulation. These simulation results are presented in Appendix A (Figure A1, Figure A2, Figure A3, Figure A4, Figure A5, Figure A6 and Figure A7, respectively, for the incidence angles of 30°, 35°, 40°, 45°, 50°, 55°, and 60°). The wind retrieval maximum errors were 0.73 m/s and 5.6° at *θ* = 30°, 0.7 m/s and 5.2° at *θ* = 35°, 0.64 m/s and 4.5° at *θ* = 40°, 0.58 m/s and 4.6° at *θ* = 45°, 0.53 m/s and 3.8° at *θ* = 50°, 0.52 m/s and 4.7° at *θ* = 55°, and 0.51 m/s and 4.0° at *θ* = 60°, respectively. The comparative results are shown in Figure 3. They demonstrate that the maximum wind speed error decreased with an increase in the incidence angle. The maximum wind direction error also tended to decrease with an increase in the incidence angle.

It was expected that the higher number of incidence angles used at the wind retrieval should decrease the wind retrieval errors as the whole number of NRCS samples would be available in this case compared with the case when only one incidence angle was used at the wind retrieval. Therefore, we considered wind retrieval in other cases when the measured NRCSs at several incidence angles in the same plane were used simultaneously. The simulations of these cases were performed using the system of Equation (4).

**Figure A8:**
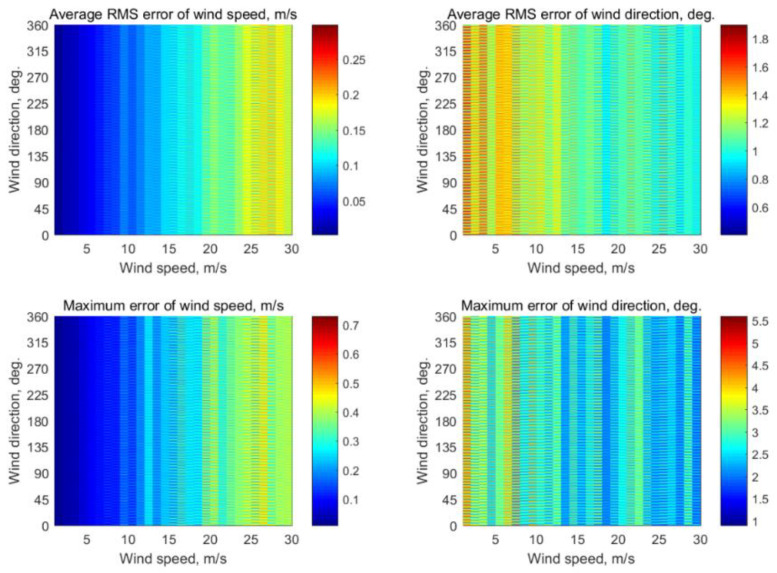
Simulation results of the use of two incidence angles of 30° and 35° for wind retrieval.

**Figure A9:**
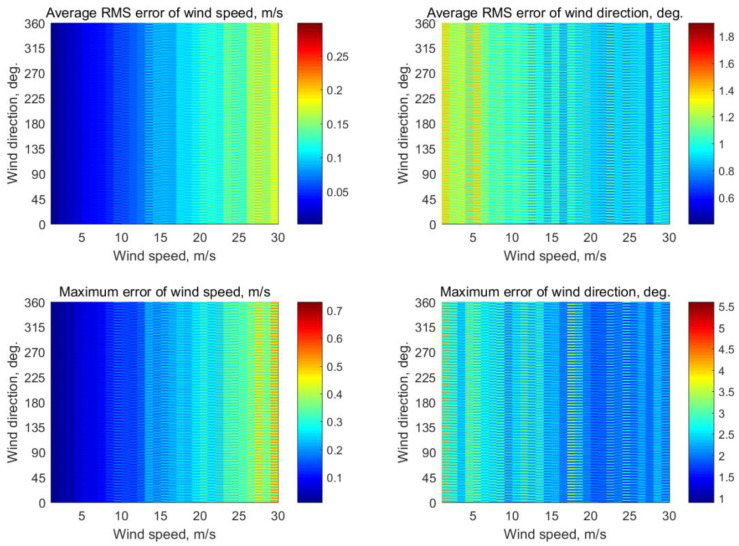
Simulation results of the use of two incidence angles of 35° and 40° for wind retrieval.

**Figure A10:**
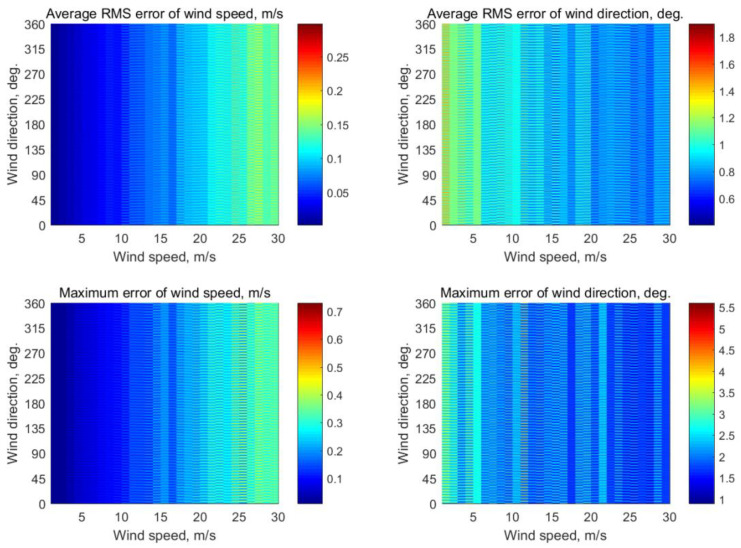
Simulation results of the use of two incidence angles of 40° and 45° for wind retrieval.

**Figure A11:**
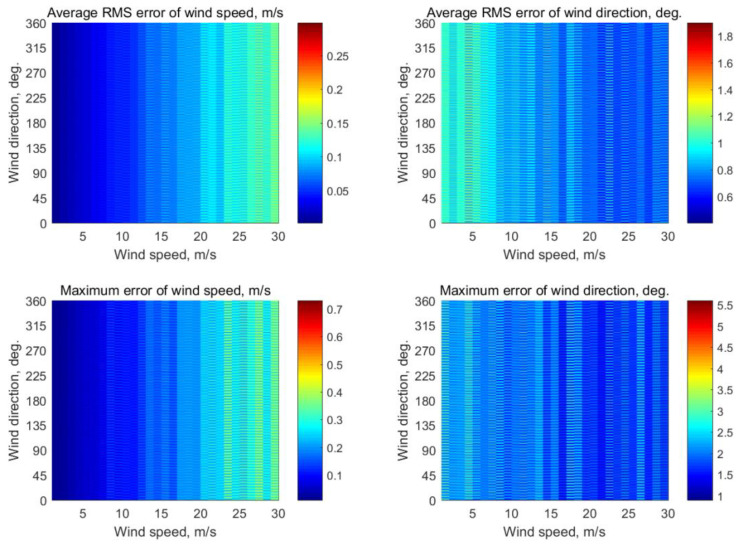
Simulation results of the use of two incidence angles of 45° and 50° for wind retrieval.

**Figure A12:**
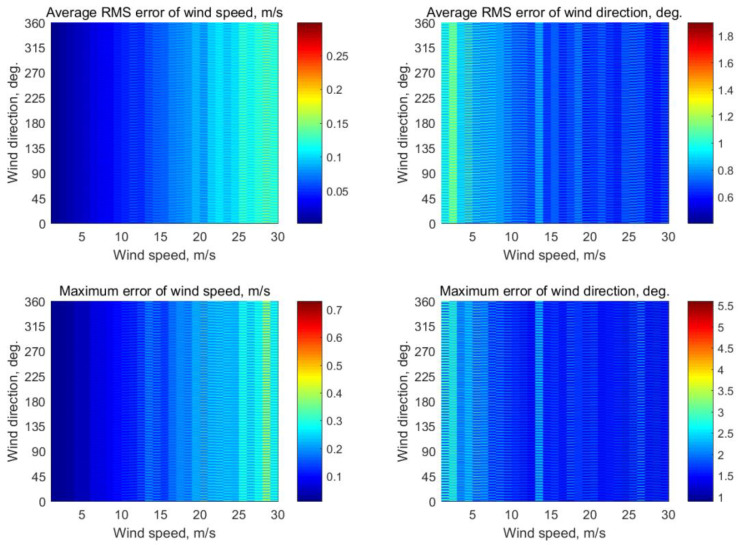
Simulation results of the use of two incidence angles of 50° and 55° for wind retrieval.

**Figure A13:**
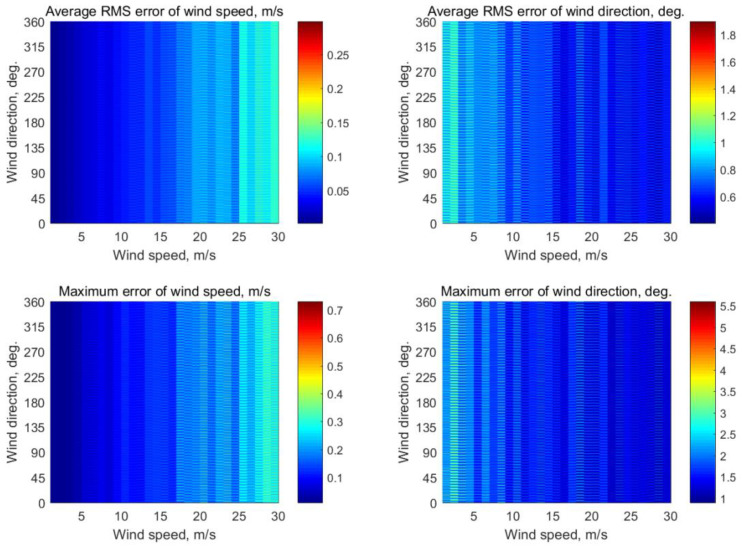
Simulation results of the use of two incidence angles of 55° and 60° for wind retrieval.

The simulation results in the case of two neighboring incidence angles used for wind retrieval are presented in Appendix B (Figure A8, Figure A9, Figure A10, Figure A11, Figure A12 and Figure A13, respectively, for the combinations of the incidence angles of 30° and 35°; 35° and 40°; 40° and 45°; 45° and 50°; 50° and 55°; and 55° and 60°. The maximum errors of the wind estimation in cases of two incidence angles are 0.53 m/s and 5.2° at *θ* = (30°, 35°), 0.54 m/s and 4.7° at *θ* = (35°, 40°), 0.47 m/s and 3.8° at *θ* = (40°, 45°), 0.42 m/s and 3.3° at *θ* = (45°, 50°), 0.41 m/s and 3.2° at *θ* = (50°, 55°), and 0.36 m/s and 3.6° at *θ* = (55°, 60°), respectively.

**Figure A14:**
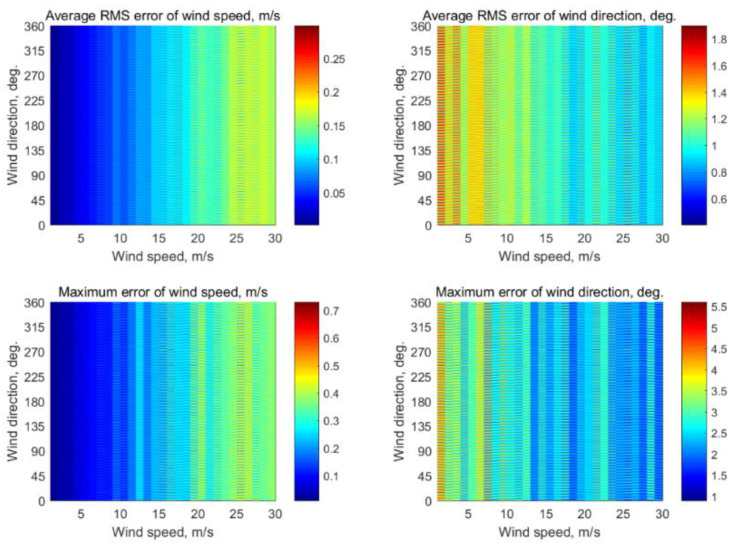
Simulation results of the use of three incidence angles of 30°, 35°, and 40° for wind retrieval.

**Figure A15:**
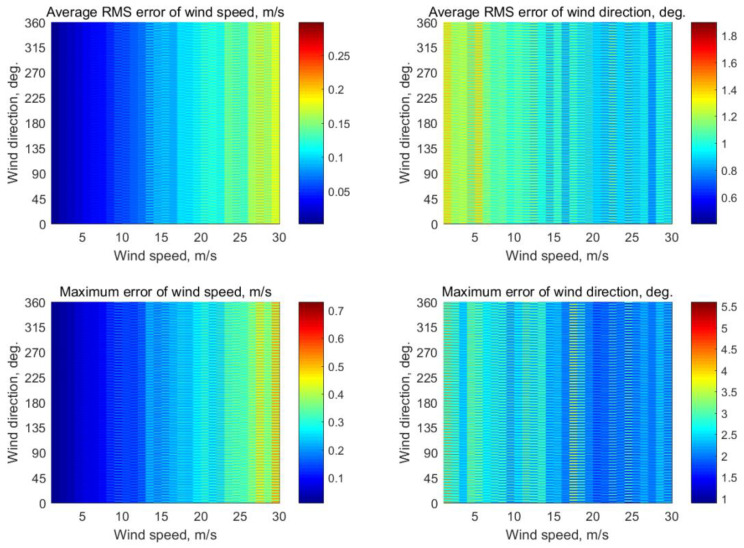
Simulation results of the use of three incidence angles of 35°, 40°, and 45° for wind retrieval.

**Figure A16:**
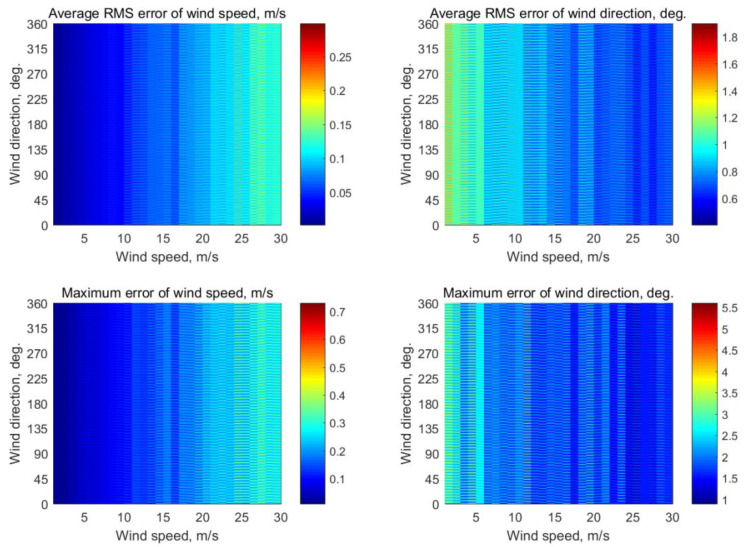
Simulation results of the use of three incidence angles of 40°, 45°, and 50° for wind retrieval.

**Figure A17:**
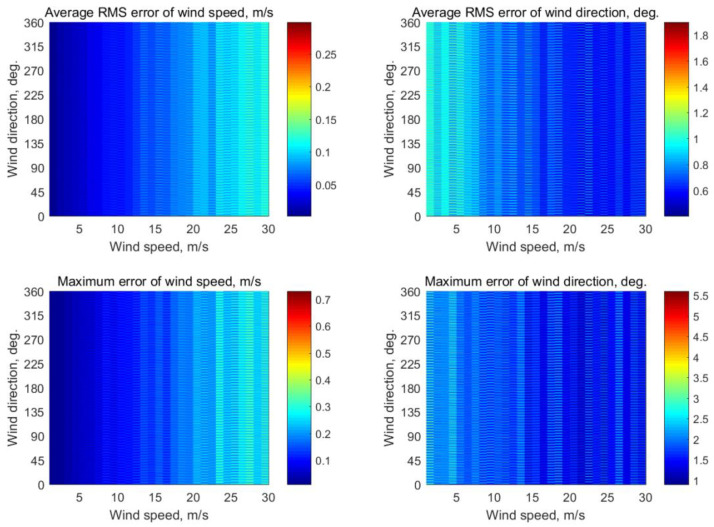
Simulation results of the use of three incidence angles of 45°, 50°, and 55° for wind retrieval.

**Figure A18:**
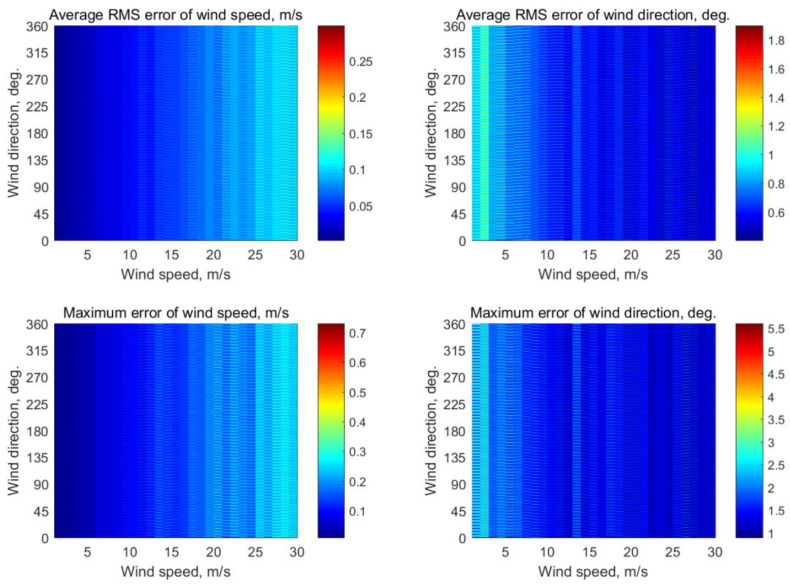
Simulation results of the use of three incidence angles of 50°, 55°, and 60° for wind retrieval.

The results obtained in the case of three neighboring incidence angles for wind retrieval are presented in Appendix C (Figure A14, Figure A15, Figure A16, Figure A17 and Figure A18, respectively, for the combinations of the incidence angles of 30°, 35°, and 40°; 35°, 40°, and 45°; 40°, 45°, and 50°; 45°, 50°, and 55°; and 50°, 55°, and 60°). The maximum errors of the wind speed and direction retrieval in cases of three incidence angles are 0.49 m/s and 5.1° at *θ* = (30°, 35°, 40°), 0.54 m/s and 4.7° at *θ* = (35°, 40°, 45°), 0.41 m/s and 3.5° at *θ* = (40°, 45°, 50°), 0.34 m/s and 3.2° at *θ* = (45°, 50°, 55°), and 0.34 m/s and 3.0° at *θ* = (50°, 55°, 60°), respectively.

**Figure A19:**
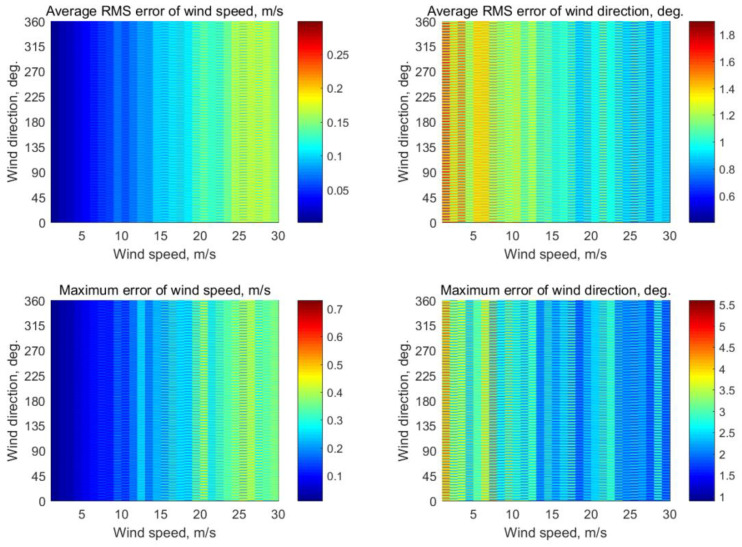
Simulation results of the use of four incidence angles of 30°, 35°, 40°, and 45° for wind retrieval.

**Figure A20:**
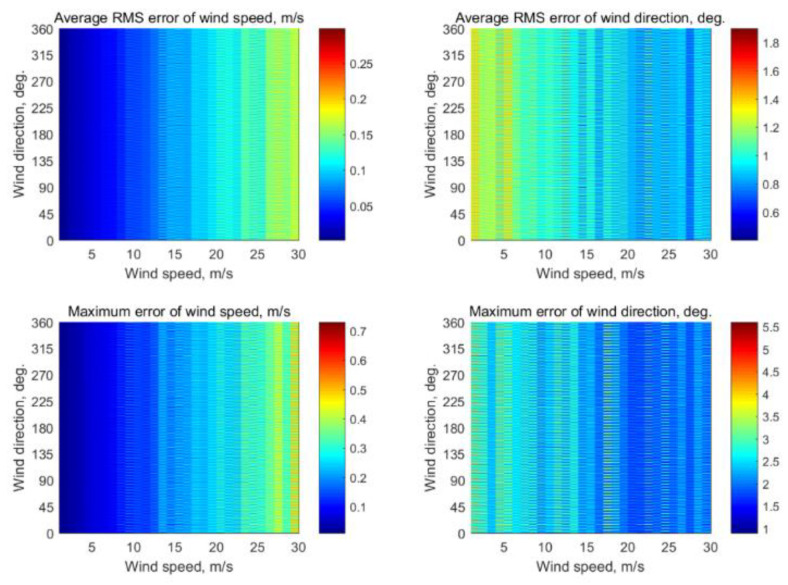
Simulation results of the use of four incidence angles of 35°, 40°, 45°, and 50° for wind retrieval.

**Figure A21:**
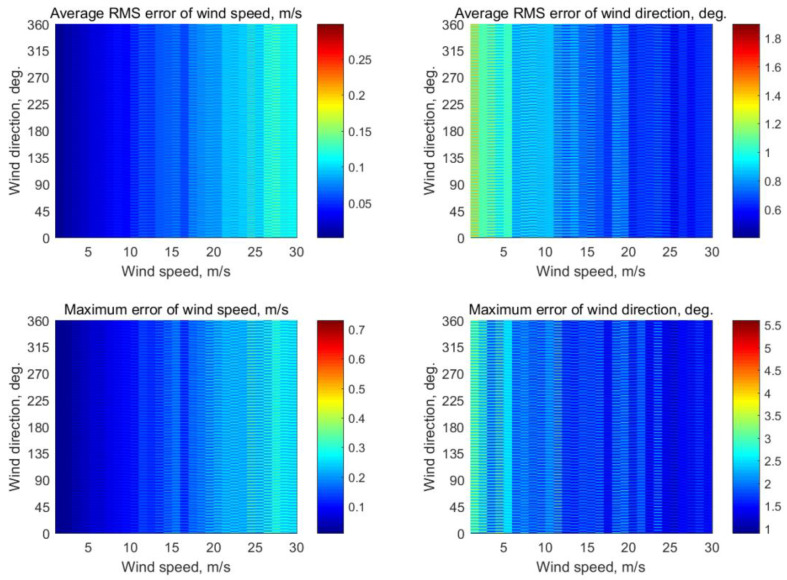
Simulation results of the use of four incidence angles of 40°, 45°, 50°, and 55° for wind retrieval.

**Figure A22:**
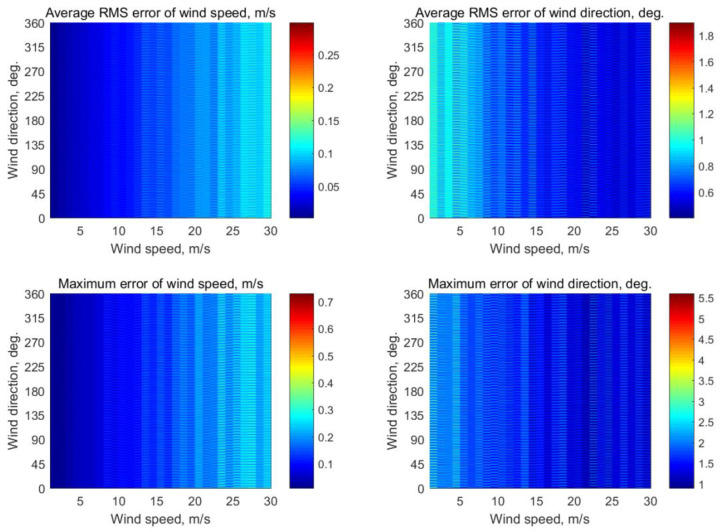
Simulation results of the use of four incidence angles of 45°, 50°, 55°, and 60° for wind retrieval.

The simulation results of when four neighboring incidence angles were used for wind retrieval are presented in Appendix D (Figure A19, Figure A20, Figure A21 and Figure A22, respectively, for the combinations of the incidence angles of 30°, 35°, 40°, and 45°; 35°, 40°, 45°, and 50°; 40°, 45°, 50°, and 55°; and 45°, 50°, 55°, and 60°. The maximum errors of the wind estimation in cases of four incidence angles are 0.47 m/s and 5.1° at *θ* = (30°, 35°, 40°, 45°), 0.51 m/s and 4.7° at *θ* = (35°, 40°, 45°, 50°), 0.37 m/s and 3.4° at *θ* = (40°, 45°, 50°, 55°), and 0.32 m/s and 3.1° at *θ* = (45°, 50°, 55°, 60°), respectively.

**Figure A23:**
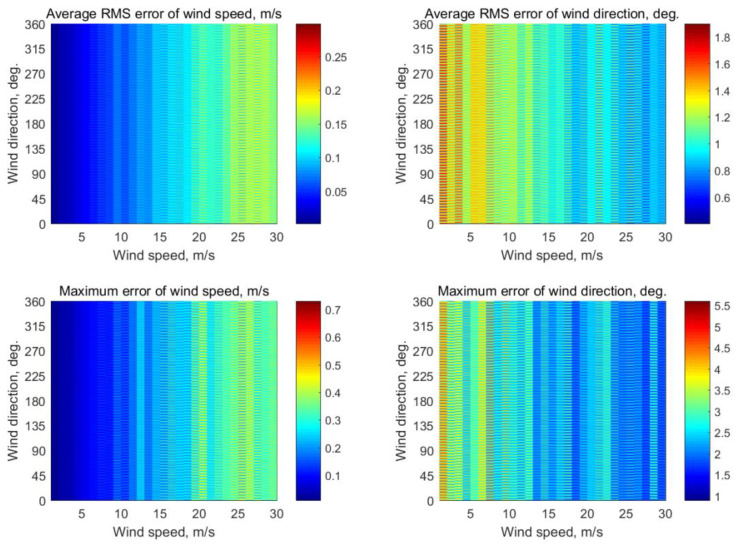
Simulation results of the use of seven incidence angles of 30°, 35°, 40°, 45°, 50°, 55°, and 60° for wind retrieval.

The simulation results of when seven neighbor incidence angles were used for wind retrieval are presented in Appendix E (Figure A23 for the incidence angles’ combination of 30°, 35°, 40°, 45°, 50°, 55°, and 60°. The maximum errors of the wind retrieval in the case of seven incidence angles are 0.46 m/s and 5.1° at *θ* = (30°, 35°, 40°, 45°, 50°, 55°, 60°).

**Figure A24:**
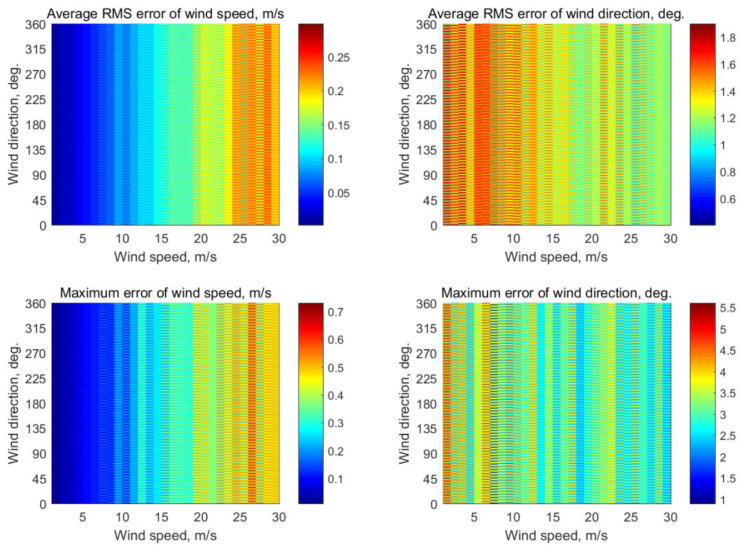
Simulation results of the use of three incidence angles of 30°, 45°, and 60° for wind retrieval.

Finally, we evaluated the maximum errors of the wind speed and direction retrieval when only three incidence angles were used but with the highest incidence angle difference of 15° between the neighboring incidence angles in the range of considered incidence angles of 30° to 60°. The simulation results are presented in Appendix F (Figure A24 for the incidence angles’ combination of 30°, 45°, and 60°. The wind retrieval maximum errors in this case are 0.69 m/s and 5.5° at *θ* = (30°, 45°, 60°).

The summarized results presented in Figure 3 clearly demonstrate that the use of NRCSs from several neighboring incidence angles provides better accuracy of the wind speed and direction retrieval than when only one incidence angle is in use. This result, of course, was expected.

The use of NRCSs from all seven incidence angles considered (30°, 35°, 40°, 45°, 50°, 55°, 60°) provides better wind speed retrieval accuracy compared to the case of only one incidence angle. At the same time, the seven-incidence-angles case does not increase the wind direction retrieval accuracy compared to the other incidence angles and their combinations in the range of the incidence angles from 40° to 60°, providing a difference of about 2°. However, the combination of seven incidence angles is not the best solution for increasing the accuracy of wind retrieval using a rotating-beam scatterometer.

Unfortunately, the use of only three incidence angles (30°, 45°, 60°) with the highest incidence angle difference of 15° between the neighboring incidence angles in the range of incidence angles of 30° to 60° showed an even worse result compared to the combination of seven incidence angles (30°, 35°, 40°, 45°, 50°, 55°, 60°).

Figure 3 demonstrates that the application of the combinations of two, three, and four incidence angles (excluding the case of three incidence angles at *θ* = (30°, 45°, 60°)) reduces the error in the wind speed and direction retrieval. The lowest value of the maximum wind speed errors is achieved with the combinations of three and four incidence angles in the range of the incidence angles from 45° to 60°. The lowest value of the maximum wind direction errors also corresponds to the numbers of the combinations of incidence angles in the same range as the incidence angles.

Nevertheless, the use of the combination of two incidence angles also demonstrates good wind retrieval accuracy compared to the case of only one incidence angle, and it is slightly worse than the accuracy achieved with the combinations of three or four incidence angles. Thus, wind retrieval within the combination of two incidence angles can be used as a simpler way to increase the wind retrieval accuracy, especially when NRCS sampling is only available near an incidence angle of 30° due to the scatterometer’s design features not allowing the application of combinations of three and four incidence angles, or the incidence angle limitation due to the size of the area observed.

The completed simulations proved that the wind retrieval errors in all the cases considered are within the typical accuracy of scatterometer wind retrieval of ±2 m/s and ±20° [43].

The area observed sets the maximum altitude limitation of airborne rotating-beam scatterometers’ applicability, as the observation circles traced on the water surface at the used incidence angles should be within this area. It is assumed that the wind and wave conditions can be considered to be the same in all parts of the area. The maximum altitude *H*_max_ of the wind retrieval method’s applicability for measuring such geometry is as follows:

(8) $$H_{\max}=\frac{D_{\max}}{2\tan\theta },$$

where *D*_max_ is the maximum diameter of the observed circular NRCS curve, which is assumed to provide the identity of the wind and wave conditions within the area of interest at the given incidence angle. For example, if the dimensions of such an area are about 15–20 km, the maximum altitudes of applicability of the considered method for the wind recovery are about 5.77 km and 17.3 km at incidence angles of 60° and 30°, respectively. Otherwise, at higher altitudes, the diameter of the observed circular NRCS curve will exceed 20 km, breaking the condition of the wind and wave identity in the observed area.

Taking this into account and applying the incidence angle step of 5° for the beams or selected cells starting with a 30° incidence angle, the maximum altitude limitations for the combinations of 1, 2, 3, and 4 incidence angles are 17.3, 14.2, 11.9, and 10 km, respectively. The lowest value of the maximum altitude limitation of 5.77 km corresponds to the case of the combination of seven incidence angles (30°, 35°, 40°, 45°, 50°, 55°, 60°) and the case of three incidence angles (30°, 45°, 60°), with the highest incidence angle difference of 15° between the neighbor incidence angles in the range of incidence angles of 30° to 60°.

Hence, the optimization of NRCS sampling during sea wind measurement using an airborne rotating-beam scatterometer mounted at the bottom or under the fuselage to increase the accuracy of the wind retrieval depends on the given altitude of measurements. If the measurement altitude requirement is only about 5.77 km, the best wind retrieval accuracy is achieved when the incidence angle or its combinations tend to the value of 60° and the combinations of three or four incident angles are used. If a higher measurement altitude is required, the incidence angle or its combinations need to be decreased properly, but this will lead to a decrease in the accuracy of the wind measurement (Figure 3). The simplest way to increase the wind measurement accuracy while providing the almost maximum altitude of measurement is to use the combination of two incident angles, as it provides lower wind speed retrieval error compared to the case of only one incidence angle, and the wind retrieval errors in the case of the combination of two incidence angles are only slightly higher than the errors generated by the use of the combinations of three or four incidence angles.

This study considered the circular NRCS sampling procedure and wind retrieval in the Ku-band. The scope of future research is the consideration of other NRCS sampling schemes in this and other bands for further improvement of the sea wind retrieval accuracy and to increase the maximum altitude of the method’s applicability.

## 4. Conclusions

Analysis of wind measurement using an airborne scatterometer with a multibeam or fan-beam rotating-antenna installed at the bottom or under the fuselage showed that in the case of only one incidence angle for wind retrieval, the wind speed error decreased with an increase in the incidence angle and the wind direction error tended to decrease with an increase in the incidence angle. This case provided the highest value of the maximum altitude of the method’s applicability for wind retrieval.

The use of NRCSs from several neighboring incidence angles allowed a better accuracy of the wind vector retrieval to be achieved compared to the case of only one incidence angle. The performed simulations showed that the use of the combinations of three and four incidence angles provided the lowest maximum wind speed errors in the range of incidence angles from 45° to 60°. The same result was also achieved regarding the wind direction errors of the combinations of incidence angles in this range of incidence angles. The maximum altitudes of the wind retrieval method with the combinations of three and four incidence angles were lower than in the cases of one incidence angle and two incidence angles.

At the same time, the wind retrieval errors in the case of the combination two incidence angles were only slightly higher than the errors generated with the use of the combinations of three or four incidence angles. However, in this case, the wind retrieval errors were lower than in the case of only one incidence angle. Moreover, this case can be used as a simpler way to decrease wind retrieval errors, especially for measurement near an incidence angle of 30°, when the scatterometer design features exclude the application of the combinations of three and four incidence angles, providing nearly the highest value of the maximum altitude of the applicability of the wind retrieval method.

Unfortunately, wind measurement using a rotating-beam scatterometer in the case of seven incidence angles was not the best solution to reducing wind retrieval errors. However, it provides at least a lower wind speed retrieval error compared to the case of only one incidence angle. Moreover, the combination of seven incidence angles is characterized by the lowest value of the maximum altitude of the wind retrieval method’s applicability.

The combination of three incidence angles with the highest incidence angle difference of 15° between the neighboring incidence angles in the range of incidence angles of 30° to 60° also demonstrated the worst result. It provided a lower wind speed retrieval error compared to the case of only one incidence angle at 30° and 35°, and a lower wind direction retrieval error compared to the case of only one incidence angle at 30°. This case was also characterized by the lowest value of the maximum altitude of the wind retrieval method’s applicability.

The errors of the wind vector retrieval with the help of the proposed wind algorithms in all considered cases of the rotating-beam scatterometers were within the ranges of a typical scatterometer’s accuracy of ±2 m/s and ±20°.

Thus, the use of several neighboring incidence angles during sea-wind measurement with airborne scatterometers or multimode radars operating in the scatterometer mode provides better wind vector retrieval accuracy compared with the case of a single incidence angle. The obtained results can be used for optimization of the NRCS sampling procedure over the ocean and sea using a rotating-beam scatterometer and for the development of new sea wind sensors or enhancement of the functionality of existing airborne maritime/ground surveillance radars, extending their application possibilities to joint and standalone measurements in oceanography, meteorology, and navigation.

## Data Availability

Data sharing not applicable.

## Appendix A

The simulation results of the wind retrieval with the system of Equation (2), when only one incidence angle is in use, are presented here. The results were obtained under the following conditions. The whole 360° azimuth circle observed was divided into *N* = 72 azimuth sectors (the azimuth sector width is 5°) at the directions of 0°, 5°, 10°, …, 355° relative to the aircraft flight direction assuming 0.2 dB instrumental noise and 87 integrated NRCS samples for each azimuth sector at wind speeds of 2–30 m/s. The results obtained for the incidence angles of 30°, 35°, 40°, 45°, 50°, 55°, and 60° are presented in Figure A1, Figure A2, Figure A3, Figure A4, Figure A5, Figure A6 and Figure A7, respectively.

## Appendix B

The simulation results of the wind retrieval with the system of Equation (4), when only two neighboring incidence angles are in use, are presented here. The results were obtained under the following conditions: The whole 360° azimuth circle observed was divided into *N* = 72 azimuth sectors (the azimuth sector width is 5°) in the directions of 0°, 5°, 10°, …, 355° relative to the aircraft flight direction assuming 0.2 dB instrumental noise and 87 integrated NRCS samples for each azimuth sector at wind speeds of 2–30 m/s. The results obtained for the incidence angle doublets of *θ* = (30°, 35°), *θ* = (35°, 40°), *θ* = (40°, 45°), *θ* = (45°, 50°), *θ* = (50°, 55°), and *θ* = (55°, 60°) are presented in Figure A8, Figure A9, Figure A10, Figure A11, Figure A12 and Figure A13, respectively.

## Appendix C

The simulation results of the wind retrieval with the system of Equation (4), when only three neighboring incidence angles are in use, are presented here. The results were obtained under the following conditions. The whole 360° azimuth circle observed was divided into *N* = 72 azimuth sectors (the azimuth sector width is 5°) in the directions of 0°, 5°, 10°, …, 355° relative to the aircraft flight direction assuming 0.2 dB instrumental noise and 87 integrated NRCS samples for each azimuth sector at wind speeds of 2–30 m/s. The results obtained for the incidence angle triplets of *θ* = (30°, 35°, 40°), *θ* = (35°, 40°, 45°), *θ* = (40°, 45°, 50°), *θ* = (45°, 50°, 55°), and *θ* = (50°, 55°, 60°) are presented in Figure A14, Figure A15, Figure A16, Figure A17 and Figure A18, respectively.

## Appendix D

The simulation results of wind retrieval with the system of Equation (4), when only four neighboring incidence angles are in use, are presented here. The results were obtained under the following conditions: The whole 360° azimuth circle observed was divided into *N* = 72 azimuth sectors (the azimuth sector width is 5°) in the directions of 0°, 5°, 10°, …, 355° relative to the aircraft flight direction assuming 0.2 dB instrumental noise and 87 integrated NRCS samples for each azimuth sector at wind speeds of 2–30 m/s. The results obtained for the incidence angle quartets of *θ* = (30°, 35°, 40°, 45°), *θ* = (35°, 40°, 45°, 50°), *θ* = (40°, 45°, 50°, 55°), and *θ* = (45°, 50°, 55°, 60°) are presented in Figure A19, Figure A20, Figure A21 and Figure A22, respectively.

## Appendix E

The simulation results of wind retrieval with the system of Equation (4), when seven neighboring incidence angles are in use, are presented here. The results were obtained under the following conditions: The whole 360° azimuth circle observed was divided into *N* = 72 azimuth sectors (the azimuth sector width is 5°) in the directions of 0°, 5°, 10°, …, 355° relative to the aircraft flight direction assuming 0.2 dB instrumental noise and 87 integrated NRCS samples for each azimuth sector at wind speeds of 2–30 m/s. The results obtained for the incidence angles of *θ* = (30°, 35°, 40°, 45°, 50°, 55°, 60°) are presented in Figure A23.

## Appendix F

The simulation results of wind retrieval with the system of Equation (4), when only three incidence angles are in use but with the highest incidence angle difference of 15° between the neighboring incidence angles (in the range of considered incidence angles of 30° to 60°), are presented here. The results were obtained under the following conditions: The whole 360° azimuth circle observed was divided into *N* = 72 azimuth sectors (the azimuth sector width is 5°) in the directions of 0°, 5°, 10°, …, 355° relative to the aircraft flight direction assuming 0.2 dB instrumental noise and 87 integrated NRCS samples for each azimuth sector at wind speeds of 2–30 m/s. The results obtained for the incidence angles of *θ* = (30°, 45°, 60°) are presented in Figure A24.
